# Sharks Violate Rensch's Rule for Sexual Size Dimorphism

**DOI:** 10.1093/iob/obae025

**Published:** 2024-07-04

**Authors:** J H Gayford, P C Sternes

**Affiliations:** Department of Life Sciences, Silwood Park Campus, Imperial College London, SW7 2AZ, London, UK; Shark Measurements, SW11 3RT, London, UK; Department of Evolution, Ecology and Organismal Biology, University of California, Riverside, CA 92521, USA

## Abstract

Systematic trends in body size variation exist in a multitude of vertebrate radiations, however their underlying ecological and evolutionary causes remain poorly understood. Rensch's rule describes one such trend—in which the scaling of sexual size dimorphism (SSD) depends on which sex is larger. Where SSD is male-biased, SSD should scale hyperallometrically, as opposed to hypoallometrically where SSD is female-biased. The evidence for Rensch's rule is mixed, and comes from a small subset of total vertebrate diversity. We conducted the first empirical test of Rensch's rule in sharks, seeking to confirm or refute a long-hypothesied trend. We find that sharks violate Rensch's rule, as the magnitude of SSD increases with body size despite sharks predominantly exhibiting female-biased SSD. This adds to a growing literature of vertebrate clades that appear not to follow Rensch's rule, suggesting the absence of a single, conserved scaling trend for SSD amongst vertebrates. It is likely that selection associated with fecundity results in the “inverse Rensch's rule” observed in sharks, although additional studies will be required to fully reveal the factors underlying SSD variation in this clade.

## Introduction

Sexual size dimorphism (hereafter SSD) occurs where body size differs between the sexes, and is widespread across animal diversity (Parker 1992; Shine 1994; Monnet and Cherry 2002; Horne et al. 2020; Krasnov et al. 2023). Both male-biased (where males are larger than females) and female-biased (where females are larger than males) SSD are observed in various species (Isaac 2005; Webb and Freckleton 2007; Rohner et al. 2018; Adams et al. 2020; Liang et al. 2022), whereas in others SSD is broadly absent (Particularly small-bodied mammals, see Lu et al. 2014 and Lindenfors et al. 2007). Historically the direction of SSD has been attributed to the balance of sexual selection for increased male size and fecundity selection for increased female size (Fairbairn et al. 2007), although the role of ecological selection in generating SSD is increasingly being recognized (Shine 1989; Mysterud 2000; Bauld et al. 2022; Bravo et al. 2024). Variation in the direction and magnitude of SSD is present not only between species, but across ontogeny (Abouheif and Fairbairn 1997; Fairbairn 1997; Dale et al. 2007). Allometry in SSD forms the basis of Rensch's rule, a hypothesized trend stating that where SSD is male-biased, the magnitude of SSD will scale hyperallometrically (becoming disproportionately larger) with body size and that where SSD is female-biased the magnitude of SSD will scale hypoallometrically, becoming disproportionately smaller with body size (Badyaev 2002; Dale et al. 2007). Comparative phylogenetic studies have found mixed support for Rensch's rule, with some suggesting the rule to be a significant, general trend (Abouheif and Fairbairn 1997 and others suggesting that it is valid only in clades dominated by male-biased SSD (Webb and Freckleton 2007). For example, Rensch's rule is violated in several large vertebrate clades that display predominantly female-biased SSD, including anurans and lizards (Liao et al. 2013; Liang et al. 2022). Intriguingly, recent studies also suggest that Rensch's rule can manifest in traits other than body size, including male ornaments and weapons (Machado et al. 2021; Reyes-Puig et al. 2023). Existing studies omit large portions of extant diversity however, and thus it remains unknown the true extent to which Rensch's rule can be generalized across phylogenetically disparate clades.

Elasmobranchii (sharks and rays) is one major vertebrate clade in which the validity of Rensch's rule has not been tested. SSD is widespread in elasmobranchs and appears to be predominantly female-biased (Conrath and Musick 2012; Gayford 2023; Gayford and Sternes 2024), although there is evidence that male-biased SSD has evolved multiple times independently within the clade (Gayford and Sternes 2024). Gayford and Sternes (2024) recently performed a comparative phylogenetic analysis of SSD in sharks, finding support for the hypothesis that differences in reproductive mode underlie the phylogenetic distribution of SSD in the clade (Sims 2005; Colonello et al. 2007; Colonello et al. 2020; Gayford and Sternes 2024). This result is indicative of spatiotemporal variation in reproductive effort modulating selection on female body size (Gayford and Sternes 2024). To date, no study has investigated (directly or indirectly) the potential for associations between SSD and body size in Elasmobranchii. Given the phylogenetic position of Elasmobranchii as the sister clade to all other jawed vertebrates (Heinicke et al. 2009) and the diversity (both taxonomic and ecological) exhibited within the clade (Compagno 1990; Heithaus et al. 2010; Heupel et al. 2014; Weigmann 2016), the validity of Rensch's rule or lack thereof in Elasmobranchii has potentially significant consequences for our understanding of SSD evolution in vertebrates more broadly.

In this study, we use Gayford and Sternes (2024)’s SSD database to test for consistent allometric trends in SSD in sharks at multiple ontogenetic stages, and consequently test the validity of Rensch's rule in selachians. This is the first formal investigation of Rensch's rule or SSD allometry in Chondrichthyes and amongst the first to apply a multi-species, comparative approach to studying SSD allometry in fish. Despite being a relatively simple analysis, testing for the validity of Rensch's rule is an important step toward understanding the evolutionary basis of SSD in diverse vertebrate clades. We predict that as a radiation with predominantly female-biased SSD, Rensch's rule will be violated in sharks. Our results have implications for our understanding of body size evolution in sharks and the extent to which SSD scales uniformly with body size across vertebrate phylogeny.

## Methodology

### Data collection

**Fig. 1:**
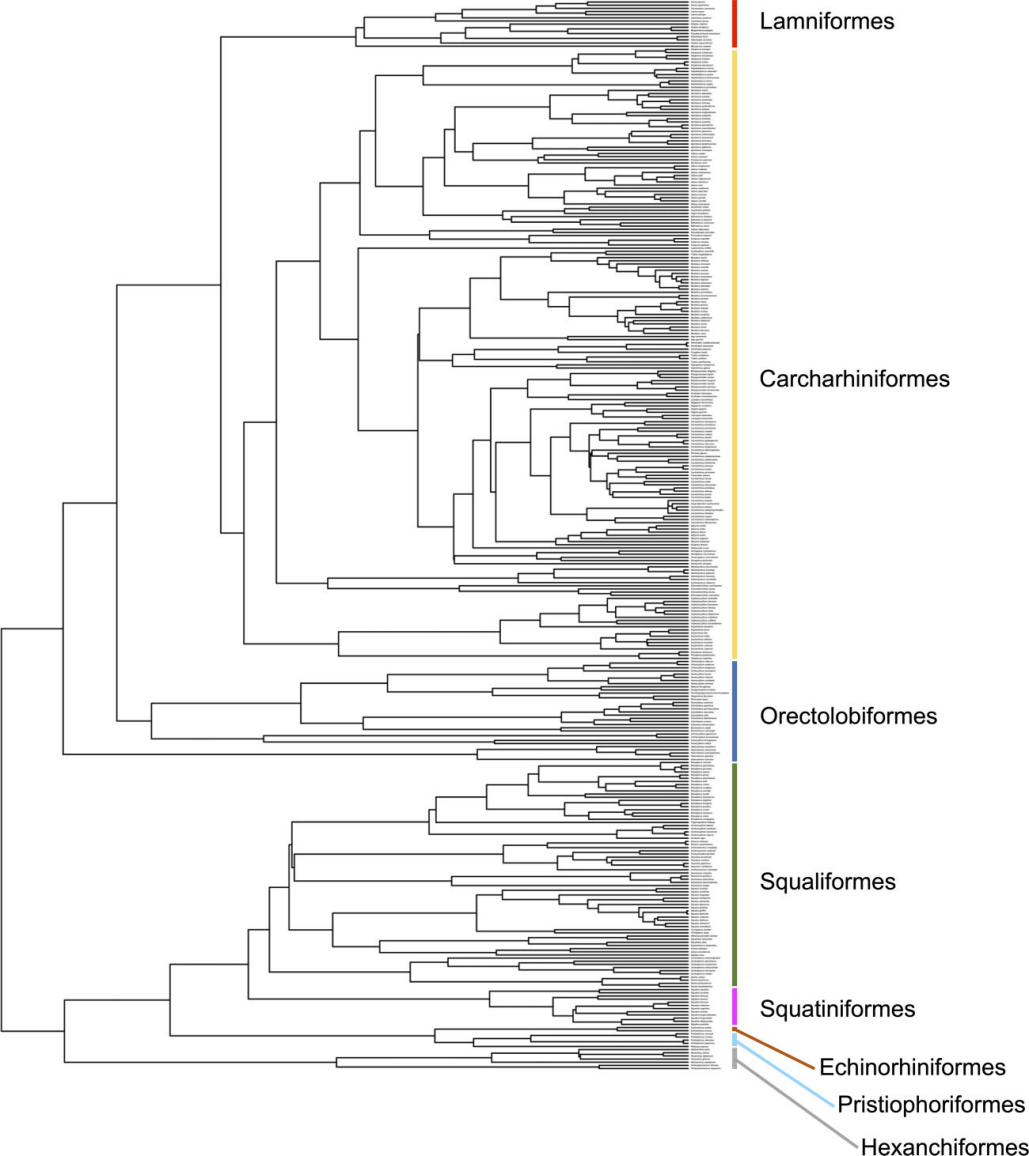
Time-scaled molecular phylogeny displaying phylogenetic interrelationships between the taxa included in this study. Branch lengths were obtained from Stein et al. (2018).

Data regarding SSD and body size in sharks were extracted from Gayford and Sternes (2024). Specifically, 2 measures of SSD were included in this study: SD% (percentage of maximum total length corresponding to the difference in median length at sexual maturity between the two sexes) and MFR (the ratio of median length at sexual maturity between males and females). Consequently, SD% provides a measure of the magnitude of SSD, whereas MFR provides a measure of the direction of SSD (Gayford and Sternes 2024). Importantly, MFR values greater than 1 indicate male-biased SSD, whereas MFR values lower than 1 indicate female-biased SSD (Gayford and Sternes 2024). The pruned, time-calibrated phylogeny from Gayford and Sternes (2024), based on topology and branch lengths of Stein et al. (2018) was also obtained and used for all phylogenetically-informed analyses. This dataset includes 339 shark species including members of all major radiations (Fig. 1).

To test the validity of Rensch's rule in sharks, we also collected maximum body length (cm) and body length at birth (cm) for each species in Gayford and Sternes (2024). These data, along with the SSD values reported in the paper, originally come from the reference book “Sharks of the World: a Complete Guide” (Ebert et al. 2021). As per Gayford and Sternes (2024), where unverified size records existed, they were ignored, and where a range of values were provided for a given measurement, the median value was taken. We divided body length at birth values by maximum body length values and multiplied these by 100 to find length at birth as a percentage of maximum length. MFR, SD%, raw maximum length values, and proportional length at birth values were all log transformed prior to data analysis. Maximum body length will hereafter be referred to as “body size.” Of course there is inherent uncertainty associated with measures of maximum body length given that new size records could be discovered at any time, a factor that is compounded by the potential for indeterminate growth in sharks. However, these data still represent the best possible approximation of maximum body lengths in sharks, and are biologically valuable when viewed in the context of these limitations.

### Data analysis

All analyses were carried out in the *R* statistical environment (R Core Team 2023). To test for possible relationships between SSD and body size while accounting for phylogenetic non-independence, we performed phylogenetic generalized least squares regression (PGLS) in the packages *nlme* and *ape* (Pinheiro et al. 2017; Paradis et al. 2019). Specifically, we fit separate regressions for each possible relationship between SSD (SD% and MFR) and body size (maximum body length and body length at birth), under both Brownian Motion model and Ornstein-Uhlenbeck models of trait evolution (Paradis et al. 2019). Brownian Motion typically represents the null hypothesis in models of trait evolution, whereas we also fit PGLS models with an Ornstein-Uhnlenbeck correction as this mode of trait evolution is more consistent with natural selection acting upon SSD, and appears to better explain the phylogenetic distribution of SSD amongst sharks (Gayford and Sternes 2024). As sharks predominantly show female-biased SSD, we could expect, were Rensch's rule to be valid, that the magnitude of SSD would scale hypoallometrically (Abouheif and Fairbairn 1997). In this scenario, PGLS should recover a significant negative relationship between body size and SD%. Contrastingly, if a positive relationship (or no relationship) is recovered, hypoallometry becomes impossible, and thus we can reject Rensch's rule.

## Results

**Fig. 2:**
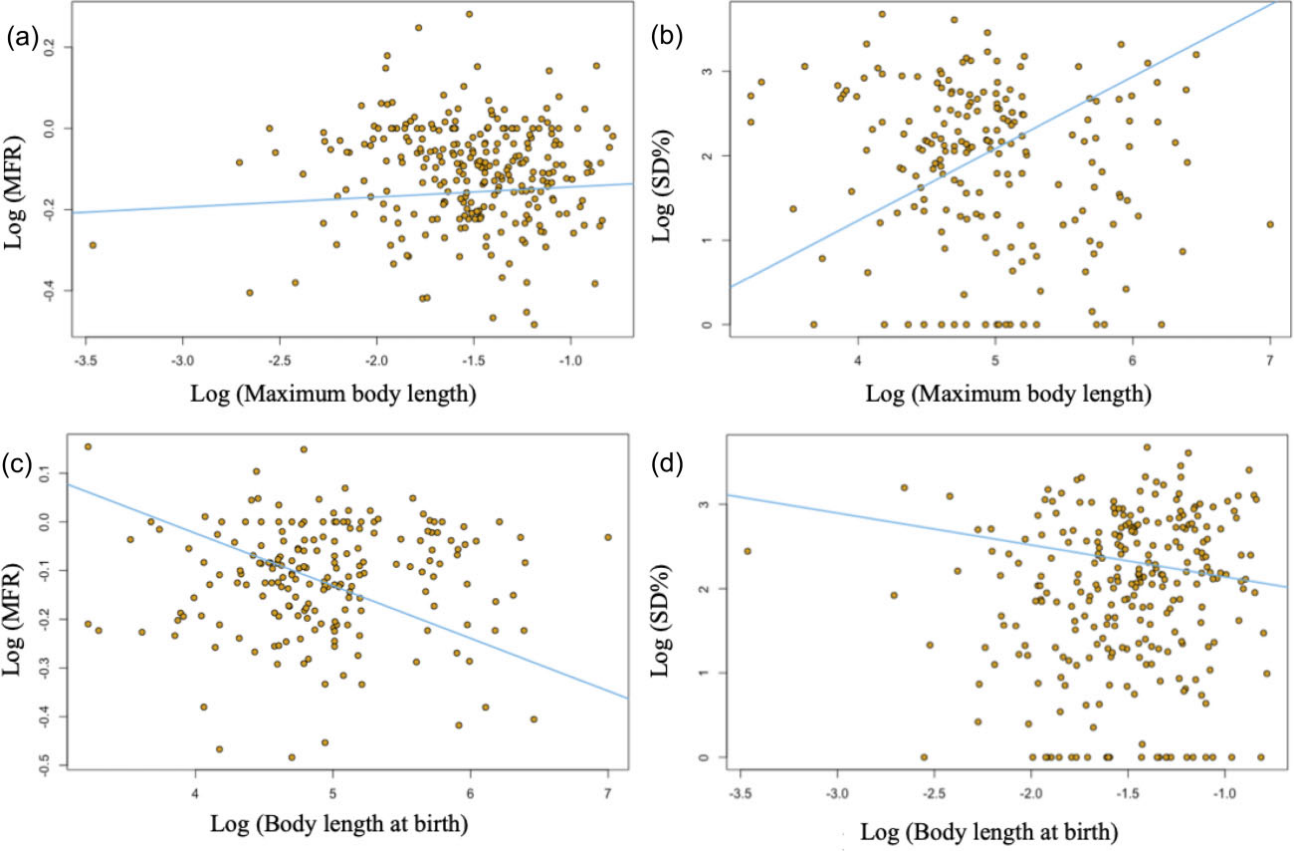
Regression relationships between maximum body length (mean maximum body length as per Ebert et al. (2021)/body length at birth and MFR/SD% obtained by PGLS analysis (assuming a Brownian Motion covariance matrix), corresponding to test statistics presented in Table 1.

PGLS analyses recovered significant relationships between maximum body length and both MFR and SD% (Table 1; Fig. 2). In the case of MFR the relationship is negative such that larger shark species show more female-biased SSD, whereas the correlation with SD% is positive such that larger shark species exhibit greater SSD (Table 1; Fig. 2). No significant relationship between SSD and body length at birth was found (Table 1).

**Table 1:** Output from PGLS analyses, including *P*-values, *T* values scaling coefficients, and standard error.

		**MFR**				**SD%**			
**Factor**	**Covariance Matrix**	**Coefficient**	***T***	***P***	**Std. error**	**Coefficient**	***T***	***P***	**Std. error**
Log (Maximum length)	BM	−0.108	4.82	<0.001	2.24e-02	0.851	4.67	<0.001	0.18
Log (Length at birth)	BM	0.025	0.90	0.37	2.77e-02	−0.378	1.74	0.08	0.22
Log (Maximum length)	OU	9.95e-04	0.81	0.94	1.23e-02	−0.19	1.92	0.06	0.10
Log (Length at birth)	OU	−4.44e-03	0.23	0.82	1.93e-02	0.257	1.74	0.08	0.15

*Note*: Covariance matrices refer to the model of trait evolution used for phylogenetic correction, where BM is Brownian Motion and OU is Ornstein–Uhlenbeck.

## Discussion

Sharks appear to violate Rensch's rule of allometry in SSD systems, further confirming the invalidity of this supposed general scaling phenomenon where female-biased SSD dominates. Rensch's rule states that in taxa with male-biased SSD, the intensity of SSD will scale hyperallometrically with body size, whereas this will not be the case where female-biased SSD is observed (Webb and Freckleton 2007; Liao et al. 2015). We found no support for the proposed rule in sharks however, with no relationship between SSD intensity and size at birth, and an either no relationship or a positive relationship (ruling out hypoallometry) between body size and SSD intensity (Fig. 2; Table 1), the opposite of what would be expected were Rensch's rule valid in this clade (Abouheif and Fairbairn 1997). These findings add to an increasing body of evidence that Rensch's rule may be valid in taxa with male-biased SSD (Abouheif and Fairbairn 1997; Fairbairn 1997), but is rarely if ever valid in taxa which exhibit female-biased SSD (Webb and Freckleton 2007; Liao et al. 2015; Zhang et al. 2023). Indeed in many taxa, including sharks, the opposite of Rensch's rule appears to be true (Fig. 2; Webb and Freckleton 2007; Halámková et al. 2013; Liao, et al. 2013; Burbrink and Futterman 2019). It is clear that in taxa with female-biased SSD, hypoallometry of SSD should be seen as the exception rather than the rule.

Several potential explanations have been posited to explain both Rensch's rule and its violation in taxa with female-biased SSD (Dale et al. 2007; Webb and Freckleton 2007; Stuart-Fox 2009; Frydlová and Frynta 2010). Most commonly, it is argued that Rensch's rule (where valid) is driven by a correlated evolutionary change in females in response to sexual selection for increased male body size (Dale et al. 2007). If this explanation is valid and generalizable, then there are at least two distinct possible explanations for the violation of Rensch's rule in taxa with female-biased SSD: sexual selection for large females (Dale et al. 2007) or relatively weak sexual selection for large males. Our understanding of sexual selection in sharks is poor, predominantly as a result of data paucity (Rowley et al. 2019), however it appears that in this clade, the latter of these two explanations is more probable. Based on what little data there is available, there appears to be no relationship between SSD and sexual selection in sharks (Gayford and Sternes 2024). Rather, variation in the spatiotemporal distribution of fecundity resulting from differences in reproductive mode appears to drive differences in SSD intensity between shark species (Gayford and Sternes 2024). Thus, it is not entirely surprising that sharks should violate Rensch's rule, given that the main requirement for hypoallometry in female-biased SSD systems (strong sexual selection for increased male body size) doesn't appear to be particularly important in driving body size differences amongst shark species.

SSD is undoubtedly abundant in nature, but there remains much controversy over its adaptive value and the selective factors influencing its evolution (Blanckenhorn 2005; Parker 1992; Reeve and Fairbairn 2001). In the case of sharks, several limitations influencing our ability to study this adaptive value include lack of fossil material and the long, indeterminate growth periods of chondrichthyan taxa. Arguably, the main barrier, however, is a lack of understanding of sexual selection: we know almost nothing about its abundance, intensity, whether it acts predominantly on males or females, or its evolutionary consequences (Gayford 2023). Given that reproductive mode is the primary determinant of SSD intensity in sharks (Gayford and Sternes 2024), future insights into SSD within this clade may seek to partition analyses into matrotrophic and oviparous groups. Indeed if, as has been speculated in past studies, sexual selection upon males is stronger in oviparous sharks (Colonello et al. 2020), we might expect Rensch's rule to be valid within certain shark lineages. Many of the most speciose shark families (Scyliorhinidae, Etmopteridae, etc.) are small in body size, potentially obscuring differential trends in SSD scaling in larger-bodied groups such as lamniform sharks. However, at a clade-wide scale, sharks clearly and unequivocally violate Rensch's rule, in line with our understanding of SSD more broadly (Dale et al. 2007; Gayford and Sternes 2024). Future studies will be required to determine the extent to which this trend is consistent across fish diversity (bony and chondrichthyan) or within specific elasmobranch clades, and the extent to which the validity of Rensch's rule depends directly upon the intensity of sexual selection in these clades.

## Data Availability

No data were generated during this study. All data utilized are fully available online and were extracted from previous studies as described in the methodology.
